# Effect of an Ankle Stabilization Strap Using a Badaging Technique on Ankle Range of Motion, Balance, and Spatiotemporal Gait Parameters in Patients with Chronic Stroke: A Randomized Controlled Trial

**DOI:** 10.3390/life15081291

**Published:** 2025-08-14

**Authors:** Sangyong Han, Taewoo Kang, Donghwan Park

**Affiliations:** 1 Department of Physical Therapy, Graduate School, College of Health Science, Kyungnam University, Changwon-si 51767, Republic of Korea; han1746@kyungnam.ac.kr; 2 Department of Physical Therapy, College of Health and Welfare, Woosuk University, Wanju-gun 55338, Republic of Korea; ktwkd123@woosuk.ac.kr

**Keywords:** ankle strap, ankle stability, balance, gait, stroke

## Abstract

Background: Elastic ankle straps are frequently used to improve ankle stability; however, they often fail to provide adequate support due to material limitations. Therefore, this study aimed to investigate the effects of an ankle stabilization strap applied using a bandaging technique on ankle range of motion, balance, and spatiotemporal gait parameters in patients with chronic stroke. Methods: Twenty-eight patients with chronic stroke were randomly assigned to either an ankle stabilization strap with bandaging technique (ASB, n = 14) group or an ankle–foot orthosis (AFO, n = 14) group. Both groups participated in treadmill gait training for 10 min per day, five days per week, for four weeks. Outcome measures included ankle dorsiflexion range of motion, total center of pressure displacement, timed up and go test, gait speed, and step length. A mixed-design analysis of variance was used for statistical analysis. Results: All outcome variables showed significant group-by-time interaction effects, and the ASB group exhibited significant within-group improvements after the intervention (*p* < 0.05). Conclusions: The ankle stabilization strap applied using a bandaging technique effectively improved ankle mobility, balance, and gait in patients with chronic stroke, suggesting its potential as a useful intervention in stroke rehabilitation.

## 1. Introduction

Stroke is a neurological disorder caused by central nervous system injury and is a leading cause of long-term functional impairments [1]. Most individuals with stroke exhibit impaired ankle dorsiflexion during the early swing phase of gait, resulting in foot drop and subsequent shortening of the plantar flexor muscles [2,3]. This muscle shortening further limits ankle dorsiflexion range of motion (DF-ROM) and overall joint mobility [4]. Reduced ankle mobility contributes significantly to abnormal balance strategies and asymmetrical gait patterns [5]. Therefore, preserving DF-ROM and restoring ankle mobility are essential goals in stroke rehabilitation [6].

Ankle–foot orthoses (AFOs) have been widely used in conventional stroke rehabilitation to support ankle dorsiflexion during the swing phase and prevent foot drop, thereby facilitating safer and more efficient gait patterns [7,8]. According to a meta-analysis conducted by Choo [8], which included 19 studies involving 434 stroke patients, AFO use significantly improved walking speed, cadence, step and stride length, and functional ambulation. Additionally, Nikamp [9] reported that AFO use positively affected ankle kinematics, cadence, stride duration, and single support duration in stroke patients, regardless of the timing of provision. However, AFOs may restrict ankle joint motion, potentially inducing abnormal muscle activation and reducing gait efficiency [10,11]. Moreover, the use of rigid plastic components often leads to discomfort and cosmetic dissatisfaction [12]. To address these limitations, elastic external supports, such as taping and elastic straps, have been proposed as alternatives to conventional orthoses [13,14]. These external supports provide joint stability while preserving mobility and have been shown to enhance balance, gait performance, and proprioceptive feedback in individuals with neuromuscular impairments [15,16]. According to Kobayashi [17], increasing the tension of a figure-eight strap applied to the ankle joint reduced plantarflexion during the early swing phase of gait in healthy adults. However, compared to rigid AFOs, elastic supports may offer insufficient mechanical stability and carry risks of secondary issues, such as skin irritation [18,19].

The bandaging technique, commonly used for immobilizing and stabilizing musculoskeletal injuries such as fractures or joint instability, involves overlapping elastic bandages by half when applying them to joints [20,21]. This technique provides stronger support and compression while allowing limited joint mobility necessary for functional movements [22]. Therefore, applying a bandaging technique to an ankle stabilization strap is expected to enhance joint stability while preserving functional ankle mobility.

Despite the potential benefits, studies investigating the effects of an ankle stabilization strap using a bandaging technique (ASB) in patients with chronic stroke remain extremely limited. Therefore, this study aimed to investigate the effects of treadmill gait training combined with ASB in patients with chronic stroke. We hypothesized that treadmill training with ASB would result in greater improvements in passive ankle dorsiflexion range of motion (DF-PROM), static and dynamic balance, and gait parameters compared to treadmill training with conventional AFOs.

## 2. Materials and Methods

### 2.1. Participants

To determine the appropriate sample size, a power analysis was conducted using G*Power software (version 3.1.2; Franz Faul, University of Kiel, Kiel, Germany) based on the results of a previous study [23]. The analysis was performed with a significance level of α = 0.05, a statistical power of 0.90, and an effect size of 0.99. The results indicated that a minimum of 11 participants per group was required. Considering a 20% dropout rate, the final sample size was set at 28 participants. The inclusion criteria were as follows: (1) individuals diagnosed with stroke more than 6 months prior, (2) individuals with stable vital signs (blood pressure, pulse, etc.), (3) individuals who were able to walk 10 m without assistive devices, and (4) individuals with a Korean Mini-Mental State Examination (K-MMSE) score of 24 or higher. The exclusion criteria were as follows: (1) a score of 3 or higher on the Modified Ashworth Scale (MAS) for ankle dorsiflexion, (2) an ankle DF-ROM ≥ 15°, and (3) current use of medications that may affect balance or gait. A recruitment notice was used to openly enlist participants among patients admitted to Happy Hospital. A total of 28 participants voluntarily agreed to participate in this study and met all inclusion and exclusion criteria; thus, all 28 were enrolled. All participants provided written informed consent after receiving a thorough explanation of the study procedures and potential risks, including pain, falls, and other safety-related issues. They were informed of their right to withdraw from this study at any time without penalty. This study was conducted in accordance with the Declaration of Helsinki and approved by the Institutional Review Board of Kyungnam University (approval number: 1040460-A-2025-011; approval date: 29 April 2025). This study was also registered with the Clinical Research Information Service (CRIS) under the identifier KCT0010582. This study followed CONSORT guidelines and was conducted according to the CONSORT 2025.

### 2.2. Experimental Procedures

**Figure 1:**
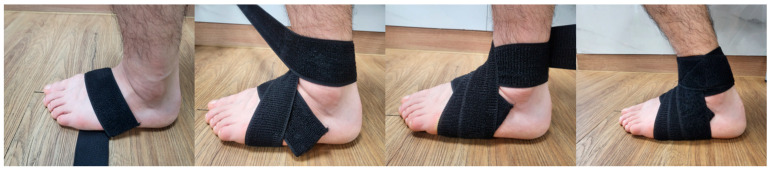
The procedure for applying the ankle stabilization strap with a bandaging technique.

**Figure 2:**
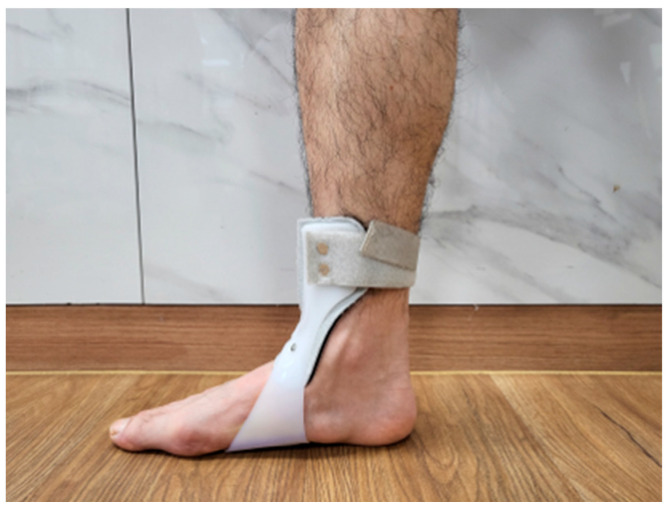
The application of the ankle–foot orthosis.

A randomized controlled trial design was used, with participants divided into two groups using an online randomization program (http://www.randomizer.org, accessed on 25 May 2025). During the allocation process, participants were numbered from 1 to 28, and a random sequence was automatically generated to assign them to their respective groups. Given the nature of the intervention, a single-blind design was employed to minimize assessment bias. The participants were assigned to either the ASB group (n = 14) or the conventional AFO group (n = 14). The ASB group performed treadmill gait training with an ankle stabilization strap using a bandaging technique (Figure 1), while the AFO group performed treadmill gait training with AFOs on the affected side (Figure 2). Both groups participated in the intervention once daily, five days per week, for a total of four weeks. To minimize confounding effects, participants were instructed to apply the ASB or AFO only during the treadmill gait training sessions. The devices were not used during other daily activities throughout the study period. Before the intervention, all participants received standardized physiotherapy sessions lasting 30 min per day. The content and duration of the exercises were strictly aligned with a predefined protocol to ensure consistency between the two groups. Each session included 10 min of range-of-motion exercises for the upper extremities, trunk, and pelvis; 10 min of weight-shifting training in both sitting and standing posture; and 10 min of over-ground gait training conducted in a rehabilitation room [24]. Baseline characteristics, including age, height, body mass, gender, affected side, stroke type, disease duration, and K-MMSE scores, were collected for all participants. Pre-intervention assessments were conducted one day prior to the intervention, and post-intervention assessments were carried out the day after the final session.

The ankle DF-ROM was measured using a goniometer (Jamar^®^, Performance Health, Warrenville, IL, USA). Balance was evaluated using both static measures (AMTI force plate, Newton, MA, USA) and dynamic measures (timed up and go test, TUG). Spatiotemporal gait parameters, including gait speed and step length, were assessed using the GAITRite system (CIR Systems, Easton, PA, USA). Each assessment, including ankle DF-ROM, static balance, dynamic balance, and spatiotemporal gait analysis, took approximately 5 min, and the total evaluation time was approximately 20 min per participant.

### 2.3. Ankle Stabilization Strap Using Bandaging Technique with Treadmill

The ASB group applied the ASB (Elastic Velcro-type strap, Happymesh Inc., Chuncheon-si, Republic of Korea) using a modified method based on a previous study [22]. (1) Participants were seated with the paretic ankle positioned at 5° of dorsiflexion. (2) The strap was initially anchored at the base of the fifth metatarsal on the dorsolateral side of the foot and directed diagonally toward the head of the first metatarsal. (3) It was then wrapped across the plantar surface to the dorsal side of the fifth metatarsal and continued diagonally toward the medial malleolus, where eversion-directed tension was applied. (4) After passing over the medial malleolus, the strap was redirected toward the first metatarsal and then wrapped across the plantar surface, encircling both the medial and lateral malleoli with 50% overlap. (5) This wrapping sequence was repeated twice more, resulting in three overlapping layers in total. Participants were then asked whether they experienced any discomfort. (6) Treadmill gait training began at a speed of 0.5 m/s and was gradually increased to a maximum of 2.0 m/s. The 10 min training session consisted of 4 min of walking followed by 1 min of rest.

### 2.4. Conventional Ankle Food Orthosis with Treadmill

The AFO group used standard, off-the-shelf U-type AFOs (UD-Flex, ADVANFIT Inc., Yatsushiro, Kumamoto, Japan) designed to maintain the ankle in a neutral position. The orthoses were available in four sizes—M/L (22.5–24.5 cm) and L (25.0–26.5 cm) for both the left and right sides—and the most appropriate size was selected for each participant. The AFO was applied at the metatarsal level and secured with a calf strap. After fitting the orthosis, treadmill gait training was conducted under the same conditions as in the ASB group, starting at a speed of 0.5 m/s and gradually increasing up to a maximum of 2.0 m/s. The total training time was 10 min, consisting of 4 min of walking followed by 1 min of rest, repeated over two cycles.

### 2.5. Outcome Measurements

#### 2.5.1. Ankle Range of Motion

Ankle DF-ROM was measured using a goniometer [25]. Participants lay prone with the knee 90° flexed, and the goniometer’s axis was aligned with the lateral malleolus. The stationary arm was positioned parallel to the fibula, while the moving arm was aligned with the fifth metatarsal. The examiner passively dorsiflexed the ankle to the point of initial resistance, measured the angle three times, and used the average value for analysis.

#### 2.5.2. Static Balance Ability

Static balance was assessed by measuring the total center of pressure (COP) displacement using an AMTI force plate [26]. The AMTI force plate, which connects to a computer via USB, has a maximum load capacity of 130 kg and dimensions of 45.5 × 502 × 502 mm. Participants stood barefoot on the force plate in a comfortable position with their eyes open, maintaining their gaze on a 15 cm visual target placed 2 m in front of them. While maintaining this posture, the total COP displacement was recorded for 30 s. For each participant, the overall COP displacement, along with sway distances in the medial–lateral and anterior–posterior directions, was analyzed. To reduce measurement errors, three trials were performed under the same conditions, and the average value was used for analysis [27].

#### 2.5.3. Dynamic Balance Ability

Dynamic balance was assessed using the timed up and go test [28]: (1) standing up from a seated position, (2) walking a distance of 3 m, (3) turning around a marker placed at the 3 m point, and returning to sit on the chair. To minimize measurement error, the test was performed three times, and the average value was used for analysis. The TUG test has excellent test–retest reliability (ICC > 0.95) and validity [29].

#### 2.5.4. Spatiotemporal Gait Parameters

Spatiotemporal gait parameters were assessed using the GAITRite system [30]. The GAITRite mat, which measures 461 cm long and 88 cm wide, contains 13,824 sensors to capture detailed gait data. To reduce measurement errors, participants were instructed to start walking 2 m before reaching the mat and to continue walking for 2 m after passing the mat’s end. Each participant completed three walking trials, and the average of these trials was used for analysis. The primary variables analyzed were gait speed and step length. The GAITRite system has shown high test–retest reliability, with intraclass correlation coefficients (ICCs) between 0.82 and 0.92 [31].

### 2.6. Statical Analysis

Statistical analysis was performed using SPSS version 25.0 (SPSS Inc., Chicago, IL, USA). The normality of data distribution was assessed using the Shapiro–Wilk test. Baseline characteristics were compared between groups using independent *t*-tests for continuous variables and chi-square tests for categorical variables. All measured variables were normally distributed (*p* > 0.05). To investigate the interaction effects between the group (ASB vs. AFO) and time (before and after the intervention), a mixed-design ANOVA was utilized. Within-group changes were analyzed using paired *t*-tests. Statistical significance was set at *p* < 0.05. Effect sizes (ESs) were calculated to evaluate the magnitude of changes between groups, with values ≤ 0.20 considered small, 0.50 considered moderate, and 0.80 or higher considered large [32].

## 3. Results

This study was conducted over a period of four weeks, and no participants dropped out. The general and clinical characteristics of the 28 participants are summarized in Table 1. No significant differences were found in the baseline variables between the ASB and AFO groups.

Table 2 shows the values obtained for all outcome measures. A mixed-design ANOVA demonstrated a significant interaction effect between group and time, suggesting that the effect of time interacted with group assignment to influence the measured outcome variables differently. Specifically, significant interaction effects were found for ankle DF-ROM (F(1, 26) = 48.76, *p* < 0.001, η^2^ = 0.652), total COP displacement (F(1, 26) = 22.23, *p* = 0.001, η^2^ = 0.368), TUG (F(1, 26) = 12.99, *p* = 0.001, η^2^ = 0.333), gait speed (F(1, 26) = 8.74, *p* = 0.007, η^2^ = 0.251), step length on the unaffected side (F(1, 26) = 42.07, *p* < 0.001, η^2^ = 0.628), and step length on the affected side (F(1, 26) = 9.20, *p* < 0.001, η^2^ = 0.396). In addition, significant main effects of time were observed for these variables. Within-group comparisons between pre- and post-intervention revealed significant improvements in both groups (*p* < 0.05). However, in the AFO group, no significant changes were found in ankle DF-ROM and total COP displacement (*p* > 0.05).

## 4. Discussion

AFOs and external supports such as taping are commonly used to assist ankle dorsiflexion in patients with stroke. However, AFOs have several limitations, including restricted ankle mobility, abnormal muscle activation, and discomfort during wear. Similarly, taping may cause skin irritation and provide insufficient mechanical stability. To address these limitations, this study investigated the effects of an elastic strap applied using a bandaging technique on functional outcomes in patients with chronic stroke. Specifically, we examined its effects on ankle ROM, balance, and spatiotemporal gait parameters. To the best of our knowledge, this is the first study to evaluate the effects of an ASB in this population. Our findings demonstrated significant group-by-time interaction effects in ankle DF-PROM, total COP displacement, TUG, gait speed, and step length. These results support the hypothesis that the ASB intervention leads to greater improvements in ankle ROM, balance, and gait performance compared to AFO use.

In the comparison of pre–post changes in ankle DF-ROM, the ASB group showed a 424% greater improvement than the AFO group. During gait, approximately 10–15° of dorsiflexion is required as the limb transitions from mid-stance to terminal stance phases [33]. This dorsiflexion movement necessitates adequate elongation of the calf muscles to allow the tibia to advance over the fixed foot [34]. Previous studies involving the application of elastic straps or kinesiology tape (KT) to the ankle have reported that maintaining dorsiflexion through external elastic support facilitates dynamic lengthening of the calf muscles and is associated with improvements in ankle ROM and balance [13,35]. In addition, the elastic properties provided by the strap may enhance ankle stability during the transition from mid-stance to terminal stance, thereby enabling sufficient elongation of the calf muscles throughout this phase [14,17]. Such repeated dorsiflexion movements during gait (especially those involving repeated lengthening of the calf muscle and tendon unit) may result in adaptive changes in muscle length [36]. Therefore, the observed improvement in ankle DF-ROM may be attributed to the elastic support provided by the ASB, which maintained the ankle in a dorsiflexed position and facilitated repetitive stretching of the calf muscle–tendon unit throughout the gait cycle.

Compared to the AFO group, the ASB group showed improvements of 254% in total COP displacement and 166% in TUG scores following the intervention. Increased mechanical stability at the ankle facilitates more effective weight-bearing on the affected lower limb during standing and walking [37]. This enhanced loading experience may augment proprioceptive input from joint and muscle receptors, thereby improving somatosensory feedback and postural control [38]. Alawna [39] demonstrated that the application of elastic bandages and KT around the ankle provides medial and lateral stability, reduced ankle instability, and enhanced proprioceptive awareness, thereby preventing further injury. Furthermore, external assistive devices such as straps, Thera-Bands, and taping can compensate for insufficient muscle strength and provide joint stability, minimizing alignment deviations during dynamic activities and facilitating muscle activation [40,41]. Shin [42] reported immediate improvements in both static and dynamic balance abilities following ankle eversion taping in stroke patients with foot drop, attributing these effects to improved ankle alignment and increased joint stability. The findings of these previous studies, along with the theoretical rationale supporting the use of external assistive devices, align with the outcomes observed in the present study. Therefore, it is possible that the ASB provided external support to the ankle joint, enhanced proprioceptive feedback, improved ankle alignment, and increased joint stability. Collectively, these effects may have contributed to the improved balance performance observed in patients with chronic stroke. Although the AFO group showed statistically significant improvements in balance-related variables, the effect sizes were small to moderate (total COP displacement: d = 0.56; TUG: d = 0.29), and the changes did not exceed the minimal clinically important difference (MCID) for TUG (3.51 s) [43], with an observed improvement of only 1.62 s. In contrast, the ASB group demonstrated large effect sizes (total COP displacement: d = 1.58; TUG: d = 1.24) and exceeded the MCID threshold (4.32 s). These findings suggest that, despite statistical significance, the clinical relevance of the AFO group’s improvement may be limited, and, thus, the results should be interpreted with caution.

Compared to the AFO group, the ASB group showed greater improvements in pre- to post-intervention changes, with increases of 184% in gait speed, 612% in step length on the unaffected side, and 303% in step length on the affected side. In this study, the application of the ASB appears to have contributed to improvements in several of these gait components. First, tension provided by the strap assisted ankle dorsiflexion during the swing phase, which prevented foot drop and promoted a more normalized swing pattern [17]. This may have led to an increase in gait speed by preventing toe dragging and reducing the compensatory hip strategies typically observed in stroke patients [3,44]. Second, the ASB enhanced ankle joint stability, potentially increasing weight-bearing capacity on the affected side during the stance phase. This prolonged support not only improved balance but also allowed for longer steps on the non-paretic side, thereby enhancing step length symmetry [45]. Liu [15] demonstrated that applying a figure-eight strap extending from the ankle to the hip in stroke patients provided ankle joint stability, improved step length, and facilitated coordinated movements of the hip, knee, and ankle joints, thus improving overall gait patterns. Furthermore, ankle dorsiflexion during terminal stance facilitates forward tibial advancement, resulting in increased passive knee flexion, which is essential for a normalized gait pattern [46]. Knee flexion during the terminal stance and pre-swing phases is positively correlated with increased ipsilateral step length due to enhanced limb advancement and foot clearance [47]. Therefore, the increased ankle DF-ROM facilitated by the ASB may have influenced knee flexion during terminal stance, subsequently resulting in increased step length on the affected side. Lastly, increased ankle DF-ROM during the stance phase may have led to a prolonged stance time and enhanced stability on the ipsilateral side, thereby increasing contralateral step length [36,48]. Although AFOs can provide stability to the ankle joint, they limit plantarflexion, thereby reducing propulsion during the push-off phase of gait [49]. In addition, the restriction of DF-ROM can shorten the stance phase duration, resulting in the decreased step length of the contralateral lower extremity [50]. Although both the ASB and AFO groups showed statistically significant improvements in step length following the intervention, the magnitude of change differed substantially between groups. In the ASB group, Cohen’s d values indicated large effect sizes for both the unaffected side (d = 0.85) and the affected side (d = 1.02). In contrast, the AFO group exhibited only negligible effect sizes (d = 0.15 and 0.18, respectively). These findings suggest that the ASB intervention was markedly more effective at improving step length compared to the AFO.

This study has several limitations. First, the participants were limited to individuals with chronic stroke who were able to walk independently. Therefore, the generalization of the findings to all stroke populations, including those in the acute or subacute stages or those with severe mobility impairments, should be made with caution. Second, the intervention period was relatively short, and no follow-up assessments were conducted. As such, the long-term effects of the intervention remain unknown. Third, although the required sample size was calculated using G*Power, the total number of participants was relatively small, which may limit the statistical power and generalizability of the results. Lastly, although identical physiotherapy protocols were applied before the intervention, their potential influence on the study outcomes cannot be completely excluded. Future studies addressing these limitations, such as including a more diverse stroke population, extending the intervention and follow-up period, and increasing the sample size, are needed to further validate and support the findings of this study.

## 5. Conclusions

In this randomized controlled trial, we investigated the effects of an ankle stabilization strap applied using a bandaging technique on ankle DF-ROM, balance, and spatiotemporal gait parameters in patients with chronic stroke. The ASB group demonstrated significantly greater improvements than the AFO group in ankle DF-ROM, static and dynamic balance, gait speed, and step length. These findings indicate that the ASB, by providing both joint stability and mobility, may serve as a clinically valuable alternative to conventional AFOs in enhancing functional mobility and gait performance during stroke rehabilitation.

## Figures and Tables

**Table 1:** General and clinical characteristics of participants at baseline (N = 28).

Characteristics	ASB Group (n = 14)	AFO Group (n = 14)	χ^2^/*p*
Age (year)	65.29 ± 3.02	65.57 ± 5.58	0.868 ^b^
Height (cm)	163.57 ± 5.14	161.00 ± 7.28	0.303 ^b^
Body mass (kg)	64.65 ± 8.91	61.09 ± 8.91	0.279 ^b^
Gender Male/Female (%)	9/5 (64.3/35.7)	7/7 (50/50)	0.445 ^a^
Affected side Right/Left (%)	8/6 (57.1/42.9)	10/4 (71.4/28.6)	0.430 ^a^
Type of stroke Infarction/Hemorrhage (%)	8/6 (57.1/42.9)	9/5 (64.3/35.7)	0.699 ^a^
Disease duration (Months)	24.14 ± 8.57	21.43 ± 7.27	0.442 ^b^
K-MMSE	26.43 ± 1.99	26.21 ± 1.53	0.752 ^b^

Abbreviations: ASB = ankle stabilization strap using bandaging technique, AFO = ankle–foot orthosis, K-MMSE = Korean Mini-Mental State Examination. Values are expressed as mean ± standard deviation or n (%): ^a^ chi-square test, ^b^ independent *t*-test.

**Table 2:** Results of statistical analysis for variables (N = 28).

Parameters		ASB Group (n = 14)					AFO Group (n = 14)					Group x Time
		Pre	Post	Change Score (CI)	*p*	ES	Pre	Post	Change Score (CI)	*p*	ES	*p*
DF-ROM (°)		6.57 ± 2.14	10.71 ± 2.02	4.14 (−4.78, −3.51)	<0.001 *	1.98	6.71 ± 1.38	7.50 ± 1.60	0.79 (−1.61, 0.04)	0.059	0.53	<0.001 ^†^
Total COP displacement (cm)		49.38 ± 2.10	45.87 ± 2.33	−3.51 (2.61, 4.41)	<0.001 *	1.58	49.76 ± 2.76	48.77 ± 3.81	−0.99 (−0.08, 2.06)	0.067	0.29	0.001 ^†^
TUG (s)		36.91 ± 2.84	32.59 ± 3.15	−4.32 (3.21, 5.44)	<0.001 *	1.43	36.31 ± 3.32	34.69 ± 4.22	−1.62 (0.44, 2.80)	0.011 *	0.42	0.001 ^†^
Gait speed (cm/s)		35.72 ± 2.41	37.37 ± 2.79	1.65 (−2.26, −1.02)	<0.001 *	0.63	36.28 ± 4.51	36.86 ± 4.97	0.58 (−1.05, −0.11)	0.020 *	0.12	0.007 ^†^
SL (cm)	unaffected	21.04 ± 2.73	23.46 ± 2.92	2.42 (−3.06, −1.78)	<0.001 *	0.85	20.68 ± 2.34	21.02 ± 2.22	0.34 (−0.96, −010)	0.020 *	0.15	<0.001 ^†^
	affected	27.23 ± 1.91	29.37 ± 2.24	2.14 (−2.85, −1.42)	<0.001 *	1.02	28.06 ± 3.01	28.59 ± 3.02	0.53 (−0.61, −0.6)	0.020 *	0.18	<0.001 ^†^

Abbreviations: ASB = ankle stabilization strap using bandaging technique, AFO = ankle–foot orthosis, DF-ROM = dorsiflexion range of motion, TUG = timed up and go test, SL = step length, CI = 95% confidence interval, change score = post-test−pretest, ES = effect size analyzed by Cohen’s D, Values are expressed as mean ± standard deviation. * Significant difference between pre- and post-intervention within the group by the paired *t*-test, ^†^ Significant differences in the interaction between group and time.

## Data Availability

Data are accessible upon request via email to the corresponding author.
